# Morphogeometric Approaches to Non-vascular Plants

**DOI:** 10.3389/fpls.2016.00916

**Published:** 2016-06-27

**Authors:** Daniel E. Stanton, Catherine Reeb

**Affiliations:** ^1^Department of Ecology, Evolution and Behavior, University of Minnesota – Twin Cities, Saint PaulMN, USA; ^2^Institut de Systématique Évolution Biodiversité UMR 7205, UPMC, MNHN, CNRS, EPHE, Muséum National d’Histoire NaturelleParis, France

**Keywords:** geometric morphology, bryophytes, liverworts, mosses, branching patterns, modularity

## Abstract

Morphometric analysis of organisms has undergone a dramatic renaissance in recent years, embracing a range of novel computational and imaging techniques to provide new approaches to phenotypic characterization. These innovations have often developed piece-meal, and may reflect the taxonomic specializations and biases of their creators. In this review, we aim to provide a brief introduction to applications and applicability of modern morphometrics to non-vascular land plants, an often overlooked but evolutionarily and ecologically important group. The scale and physiology of bryophytes (mosses, liverworts, and hornworts) differ in important and informative ways from more “traditional” model plants, and their inclusion has the potential to powerfully broaden perspectives in plant morphology. In particular we highlight three areas where the “bryophytic perspective” shows considerable inter-disciplinary potential: (i) bryophytes as models for intra-specific and inter-specific phenotypic variation, (ii) bryophyte growth-forms as areas for innovation in architectural modularity, and (iii) bryophytes as models of ecophysiological integration between organs, individuals, and stands. We suggest that advances should come from two-way dialog: the translation and adoption of techniques recently developed for vascular plants (and other organisms) to bryophytes and the use of bryophytes as model systems for the innovation of new techniques and paradigms in morphogeometric approaches.

## Introduction

Morphology and its geometric underpinnings have long formed an important part our understanding of plant biology at all scales, through Goethe’s traditional work (Goethe, 1790), Traditionally considered in a structural or systematics point of view (e.g., Sneath and Sokal, 1973; Oldeman, 1977; Hallé et al., 1978; Dickinson et al., 1987; MacLeod, 2002) highlighting of geometric patterns is now built into a dynamic approach aiming to understand biological integration and modularity in the broadest sense, at all scales and levels, from development, physiological ecology to evolution (Castellanos et al., 1989; Jones, 1993; Pigliucci, 2003; Eble, 2005; Lüttge, 2012; Murren, 2012; Ambruster et al., 2014; Klingenberg, 2014). Mathematical advances in the latter half of the 20th century, such as topological techniques for disentangling shape from size and multivariate statistics provided the groundwork for modern geometric morphology. Recent innovations in modeling and image analysis have greatly expanded the power of morphometric analyses (Kaandorp and Kübler, 2001; Le Bot et al., 2009; Schindelin et al., 2012; McKay, 2013), opening up new avenues for applications such as high-throughput phenotyping and effective canopy modeling (e.g., Araus and Cairns, 2014; Bucksch et al., 2014; Puttonen et al., 2016).

The majority of recent innovations in plant geometric and morphometric analysis have focused on seed plants (angiosperms and gymnosperms). Although this bias reflects a majority of species of economic interest, it leaves aside much the overall range in land plant morphologies and functional forms. Our objective is to provide a perspective from one of those side-lines, bryophytes, in the hope that greater attention to non-vascular plants in morphometry will not only advance studies of those overlooked groups, but also contribute novel perspectives and emphases to plant morphologists.

Although quite morphologically and taxonomically diverse (∼20,000 species worldwide; Shaw et al., 2011), evolutionarily informative (Ligrone et al., 2012) and ecologically important (Lindo and Gonzalez, 2010), bryophytes are often unmentioned in modern morphometric reviews (e.g., Jensen, 2003; Diggle, 2014). This mini-review seeks to provide an overview of recent developments in the geometric and morphometric analysis of non-vascular plants. After a brief introduction to bryophytes, we present three key scales of analysis at which closer integration between plant morphometry and bryology stands to benefit both fields: organ geometry, branching patterns, and canopy-stand integration.

## Why Bryophytes?

**FIGURE 1:**
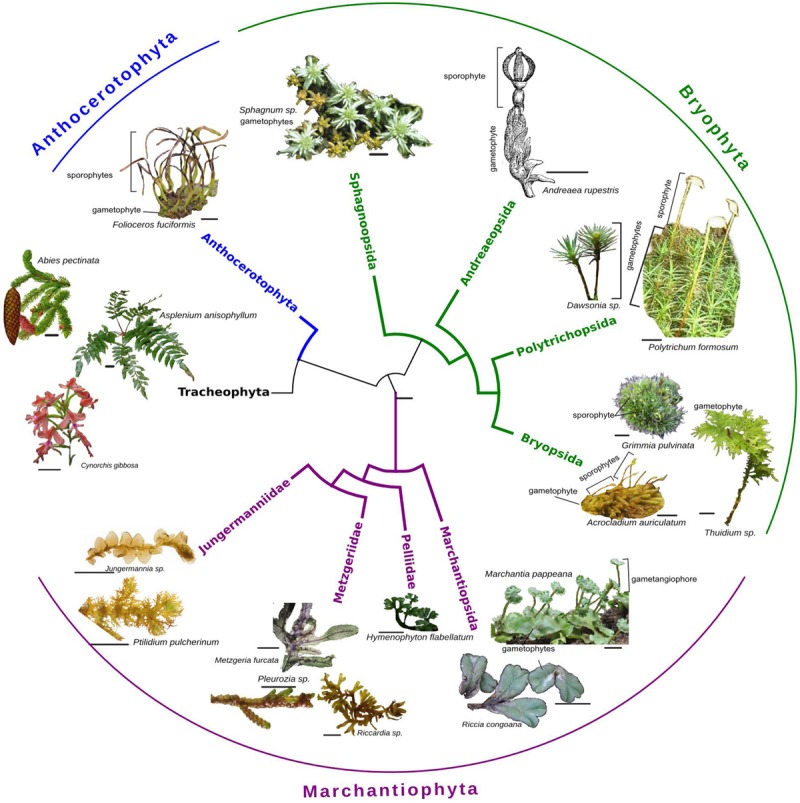
**Phylogenetic tree (adapted from Crandall-Stotler et al., 2008; Buck and Goffinet, 2009; Chang and Graham, 2011) showing the three main clades of bryophytes: liverworts (Marchantiophyta), mosses (Bryophyta), and hornworts (Anthoceratophyta) as well as their relationship to vascular plants (Tracheophyta).** The images (scale bar 1 cm) illustrate a few examples of the wide diversity of morphologies found both within and across clades. All images by CR or DS.

Bryophytes are often treated as a unit; however, they are a paraphyletic group of at most three major clades (liverworts, mosses, and hornworts) that differ greatly in their morphology and physiology (**Figure 1**). They are united primarily by the dominance of haploid (gametophytic) stages of the life-cycle and a tendency toward poikilohydry (and often dessication tolerance) rather than internal water conduction (with notable exceptions such as *Dendroligotrichum*; Hébant, 1977; Atala, 2011). The former may make them particularly plastic to genetic or environmental changes, while the latter encourages a wide array of morphological and physiological responses to water availability. Despite these differences, and a deep evolutionary history of divergence (∼450 million years), many developmental pathways are shared with other land plants (e.g., Rensing et al., 2008; Jones and Dolan, 2012; Xu et al., 2014). Indeed, several bryophyte species have a long history of use as model systems in plant biology: the thalloid liverwort *Marchantia polymorpha* and the ephemeral moss *Physcomitrella patens* in particular have been used widely in plant molecular biology and development (e.g., Prigge and Bezanilla, 2010; Bonhomme et al., 2013; Bowman et al., 2016).

All of the above provide clear motivations for closer incorporation of bryophytes into plant morphological geometry. Morphologists may find value in the wide range of morphologies presented by bryophytes, and in the challenge of accommodating a wider spectrum of plant forms. Developmental and molecular biologists will benefit from improved phenotyping techniques for these evolutionarily important model organisms. Functional ecologists have much to learn from groups in which morphology at multiple scales closely influences local micro-environment and poikilohydry tightly couples form to function. And lastly, bryologists will gain from the cross-application of novel techniques to what remains a small and understudied discipline.

### Geometries of Bryophyte Organs: Models for Inter- and Intra-Specific Variation

Bryophytes have long been known for their striking intra- as well as inter-specific variation, in particular in response to environment (e.g., Davy de Virville, 1927–1928; Birse, 1957). This plasticity is uneven: gametophytes often display a high degree of polymorphism while sporophytes remain less variable, being especially conserved among liverworts and hornworts (Schuster, 1966; Vanderpoorten and Goffinet, 2009). The causes of this high variability at the individual level can be linked to ecological, geographical and evolutionary factors (e.g., Forman, 1964; Glime and Raeymaekers, 1987; Vanderpoorten et al., 2003; Buryová and Shaw, 2005; Medina et al., 2012, 2015), and the majority of traditional morphometric studies focused on interpreting this variability.

Gametophytic plasticity presents a challenge to the development of clear and shared species delimitations; Schuster (1966) noted that “ideally, experimental data must be a frame of reference”, paving the way for experimental or integrative taxonomy (using “common gardens” of putatively different species to eliminate environmental effects; McQueen, 1991; Dayrat, 2005; Cano et al., 2006). At the peak of numerical taxonomy in 1970s–1980s, morphometry was a popular tool supporting morphological species delimitation (Hewson, 1970; Bischler-Causse, 1993), in particular helping to make decisions on species hypotheses for problematic taxa, where cryptic species may have been previously overlooked. In bryology, traditional morphometry involves measurements of gametophyte organs (leaves, stem, cells, including minute ornamentation), and sporophytes (seta, capsule, and peristome measurements). Measurements are made on living plants (Cano et al., 2006; Gonzalez-Mancebo et al., 2010; Yu et al., 2012), from single digitized images (De Luna and Gomez-Velasco, 2008), or from shallow image stacks (Renner et al., 2013b).

Today, at the inter-specific level, traditional morphometrics results analyzed from univariate or multivariate methods are compared to molecular species delimitations. For example, *Braunia andrieuxii* and *B. secunda* are discriminated by length of recurved margin and size of upper cells; the variation is always greater between species than within each species (De Luna and Gomez-Velasco, 2008) In the *Tortula subulata* complex, morphometrics recognize four species but not two of the previously known varieties (Cano et al., 2006). However, other species complexes show no clear morphological discontinuities (e.g., Vanderpoorten et al., 2003) and even greater intra- than interspecific variation (Renner et al., 2013a). Variations within populations of a single species (infra-specific level) have also been explored through traditional morphometry, showing strong correlations with geographical and ecological factors (Pereira et al., 2013). Finally, morphometrics have been incorporated into citizen science in an ongoing initiative at the Field Museum in Chicago^1^ From images of the leafy liverwort *Frullania*, the public is asked to measure leaves and lobules to build a huge dataset for analysis at intra- or inter-specific levels.

Geometric morphometry, which includes shape analysis through coordinates analysis of homologous points (landmarks; Bookstein, 1991) or outline analysis (elliptical Fourrier analysis; Ferso et al., 1985), allows to one quantify shapes and to explore their dependence on size (allometry) via combination of quantification with multivariate analysis. We will highlight two recent applications in bryology that have used geometric morphometry to unlock nuanced evolutionary understandings of form and development: liverwort lobules (Renner et al., 2010, 2013b; Renner, 2015) and moss sporophytes (Rose et al., 2016).

In the first example, the morphological variation in the lobule of the compound liverwort leaf, was linked to developmental heterochrony (Renner et al., 2013b), correlated with biogeographical and historical hypotheses (Renner, 2015). Beginning with species delimitation (including intra-specific variation; Renner et al., 2010) they broadened the issues to evolutionary questions, linking morphometrics to ontogenetic and phylogenetic analysis (Renner et al., 2013; Renner et al., 2013b; Renner, 2015). Univariate and multivariate analysis were combined with evolutionary methods: ontogenetic calibration, reconstruction of ancestral states for shape and duration of growth, reconstruction of phylomorphospace, and Bayesian analysis of macroevolutionary mixtures (BAMM). This led not only to confirmation of inter-specific variation but also an understanding of pitfalls associated with these methods: for example, only measurements of mature lobules recovered informative patterns. Furthermore, convergences in morphospace lead to testable hypotheses of functional and historical explanations of lobule morphologies (Renner, 2015).

In mosses, Rose et al. (2016) similarly combined geometric morphometric analyses (in this case harmonic amplitudes as descriptors of sporangium shape) with evolutionary hypothesis testing. They found strong correlations between shape and habitat indicative of repeated functional shifts. Shifts in sporangium shape were also found to correlate with increases in speciation rates, but not always in conjunction with shifts in habitat.

These adoptions of geometric morphometry in bryology show how several properties of bryophytes make them particularly amenable to such studies. The revival properties of bryophytes provide a constant and relatively easy availability of material, even from herbarium specimens; the usually huge number of objects in a single sample and the ease of manipulating digitalized images ensure perennial perspectives in morphometrics applications.

## Branching Patterns

Above the organ-level, a different type of morphometrics, inspired by graph theory and modularity, has proven productive. Bryophytes tend to be organized into two broad morphological types: leafy, erect or prostrate shoots with associated leaf-like flattened organs (mosses and some liverworts) and thalloid, lacking the differentiation between shoots and leaves (hornworts and some liverworts). Although outwardly quite different, both of these morphologies are based in modular development, arising from repetition of the same structure at different levels.

**FIGURE 2:**
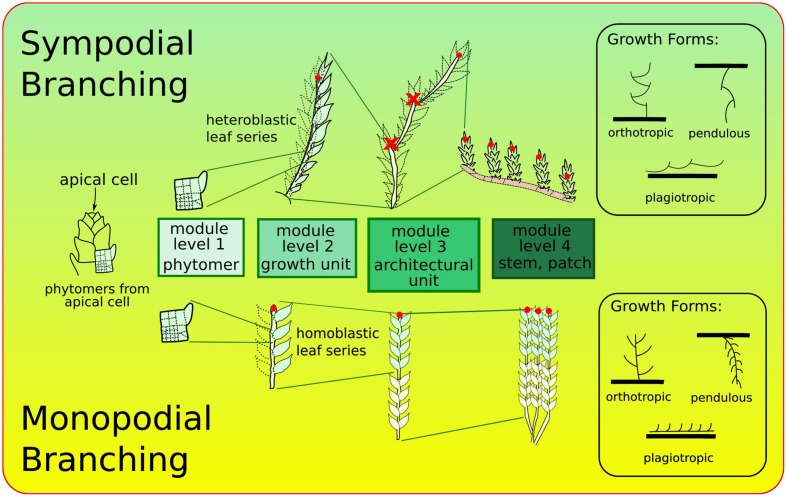
**Major branching forms in bryophytes: sympodial (top) with connected modules of same level, and monopodial (bottom).** Red dots indicate the locations of the active apical meristems at each of the levels of organization. Each of these branching forms can be found in a range of growth-forms (e.g., orthotropic, plagiotropic, etc.) and perichaetial positions (locations of sex organs, not shown). Growth and branching forms adapted from La Farge (1996).

The ontogeny of leafy bryophyte shoots, including formation of stem, leaves, and branching, has been studied for over a century (Clap, 1912; Berthier, 1970; Renzaglia, 1982; Mishler and De Luna, 1991). Branching patterns reflect ramification characteristics of shoots. They are directly linked to modularity, each ramification signaling the formation of a new module. Two types of branching patterns are recognized in bryophytes (La Farge, 1996; Goffinet and Buck, 2013): (i) sympodial, consisting of connected modules of the same level and (ii) monopodial, consisting of one module, itself connected to independent modules of different levels (**Figure 2**). Integration at the individual level, considered then as an architectural unit, defines growth-forms which depend also of perichaetial position and direction of growth (orthotropic or plagiotropic).

Modular lateral branching allows plant architecture and space filling to respond to environmental constraints, making it important to detect specific patterns (morphological modularity) in order to understand their relationship to development, environment and evolutionary history. Control of branching of the sporophyte is well known in flowering plants but conservation of the mechanisms in bryophyte gametophytes have been only briefly explored (Ashton et al., 1979; Fujita and Hasebe, 2009; Bennett et al., 2014). Using both cultivation experiments and modeling of branching patterns, Coudert et al. (2015) showed that branch initiation is patterned in the model moss *P. patens.* They highlighted regulation mechanisms by auxin, cytokinin and strigolactone. The moss shoot was represented as a connected graph with vertex and connecting edges (VVe modeling; see Abley et al., 2013); a vertex represents a metamer or an apex. Growth was simulated by periodically adding new vertices and by constraining different parameters: apical inhibition over branching via auxin; apical source of auxin, and transportation to neighboring metamers implying different concentrations in each metamer that can be calculated.

Analysis of branching patterns and morphometric characters can also be used for integrative taxonomy, for example in thalloid liverworts, in which phenotypic variability makes landmark-based morphogeometric approaches nearly impossible (Reeb, 2014). This approach was inspired by work on branched organisms, such as corals and sponges (Kaandorp, 1999; Kaandorp and Kübler, 2001; Kruszynski, 2010). The thallus is described as a connected graph, ordered using Horton–Strahler’s law (Strahler, 1952; Tarboton, 1996) and consequently each marker is defined as either vertex (junction or apex) or edge. A thallus is considered as a tree rooted by a terminal vertex of maximum order, and branches of order *n* treated as sub-trees rooted by a vertex of order *n-1*. Following these definitions, all lengths, widths, distances, and angles can be measured on a single thallus with a precise, reliable and repeatable method. Although impossible to do by eye, two software programs (2D and LeafSnake) have been developed that automatically acquire measurements from digitized images of thalli. Statistical analysis show that specific branching patterns, typical of a species or group of species, can be identified (Reeb, 2014). These approaches are complementary to those conducted on fixed animals (Kaandorp and Kübler, 2001), but also in medicine (neuronal or blood webs, e.g., Grueber et al., 2002), in geology (river catchment areas, e.g., Zanardo et al., 2013), and even in data-mining (email networks, e.g., Guimera et al., 2006) reflecting universal and mathematical laws driving such constructions.

The branching patterns of bryophytes and other early land plants are also of interest to studies of plant allometry. Some attributes of bryophyte sporophytes scale allometrically in a manner similar to vascular plants (Niklas, 1994), but this is not universal. Vascularization imposes different constraints on tissue investment during growth, and since bryophytes vary greatly in the nature and specialization of water conducting tissues (Ligrone et al., 2000), they may not all be constrained in the same manner. Interestingly, one of the tallest moss species known, the internally conducting *Dendroligotrichum dendroides*, does show branching allometries consistent with those of other vascular plants (Atala, 2011; Atala and Alfaro, 2012).

## Shoots and Canopies: Whole-Plant Approaches

A key consideration in any comparison vascular and non-vascular models of plant architecture (allometric or otherwise) is that of scale. The individual modular units of bryophytes combine to determine form and function at larger physical scales, as branching patterns are integrated at the individual level (“growth-form”) and at the population level (“life-form”). The biophysical scales at which many bryophytes operate are small enough that phyllids are not adequate analogs for vascular leaves; instead, functional analogies to vascular leaves must include some combination of leaf, shoot, and canopy properties (Waite and Sack, 2010). While this may initially seem constraining, the explicit need for cross-scale integration presages some of the current directions in vascular plant modeling, where the ecophysiological and biophysical consequences of shoot and stand structure are only beginning to be considered.

The shoot and stand scales can be particularly difficult to disentangle in bryophytes. In many bryophyte species, particularly acrocarpous mosses, the individual shoots grow tightly packed into turves or cushions. These can be multiple shoots of the same individual, closely related individuals or even multi-species mixes. This life-form modifies the impact of any given shoot architecture on light penetration, water retention and gas-exchange: in a study of 22 subarctic bryophyte species, Elumeeva et al. (2011) found that shoot density was a better predictor of water retention than anatomical properties such as cell wall thickness. Shoot density can be environmentally variable within species, and the extent of its dependence on shoot architecture remains unresolved.

In cushion-forming species of mosses, the size of the cushion alone can strongly determine physiological function, determining water balance and gas exchange (Zotz et al., 2000; Rice and Schneider, 2004). Simple geometric relationships between surface area and volume allow larger cushions to remain hydrated, and, therefore, gain carbon, for much longer periods. These scaling relationships appear to be partially species-specific; cushions can vary from hemispherical (*Leucobryum glaucum*; Rice and Schneider, 2004) to flattened (*Grimmia pulvinata*; Zotz et al., 2000). How cushion shape relates to shoot architecture and/or environment has yet to be explored more widely, despite the relative simplicity of the geometric methods required.

The surface-area relationship described by cushion size and shape is a coarse-grain simplification. The individual shoot canopies aggregate to create a rough canopy layer, analogous to that of a forest. Surface roughness has large effects on boundary-layer properties, and thus on the gas-exchange properties of a moss clump. Surface roughness has traditionally been measured by contact surface probes (Rice et al., 2001), but more efficient laser scanning methods (Rice et al., 2005) and stereoscopic image analysis (Krumnikl et al., 2010), drawing on analogies to LIDAR scanning of forests. Uptake of these methods in bryology has to date been limited (although see Acosta-Mercado et al., 2012), but their application, in conjunction with shoot- and leaf-scale architectural characterizations, shows great potential (Rice and Cornelissen, 2014).

An important distinctive feature of bryophyte canopies is that many of the surface properties are highly dynamic. Shoot and leaf structure are strongly determined by hydration state, allowing bryophytes to rapidly adjust to changing water availability. The leaves of the desert moss *Syntrichia caninervis* change angle by over 40° within seconds of rehydration, increasing exposed surface area (Wu et al., 2014). Such changes have the potential to be documented using laser-scanning approaches, as in vascular plants (Puttonen et al., 2016), providing a direct link between shoot scale dynamics and canopy surface roughness. Due to their small size, bryophytes are also particularly amenable to imaging chlorophyll fluorescence approaches (e.g., Coe et al., 2012; Stanton et al., 2014; Malenovský et al., 2015), making it possible to combine architectural imaging and photosynthetic measurement into synchronous evaluations of physiological activity.

## Promising Directions

Bryophytes offer a number of promising avenues for future research. The small size and high plasticity of bryophytes make them particularly amenable to large replication at low cost, especially as improvements in image processing allow for increasing automatization. A future expansion from two-dimensional to three-dimensional image processing and analytics will unlock applications to a wide range of organisms where branching has recently been shown to be ecologically informative, such as algae (Koehl et al., 2008; Demes et al., 2013) and lichens (Stanton and Horn, 2013; Esseen et al., 2015).

Although there have been numerous recent applications of innovative geometric approaches to bryophytes (e.g., Reeb, 2014; Rice and Cornelissen, 2014; Coudert et al., 2015; Renner, 2015; Rose et al., 2016), the field is still very young and ripe for further exploration. We suggest that advances should come from two-way dialog: the translation and adoption of techniques developed for vascular plants (and other organisms) to bryophytes and the use of bryophytes as model systems for the innovation of new techniques and paradigms in morphogeometric approaches. This will require bryologists to adopt or adapt some terms and concepts used for vascular plants, but also for researchers more familiar with vascular plants to acknowledge and incorporate the complexity of bryophyte form and function, rather than misleadingly characterizing them as “primitive and boring”.

## Author Contributions

All authors listed, have made substantial, direct and intellectual contribution to the work, and approved it for publication.

## Conflict of Interest Statement

The authors declare that the research was conducted in the absence of any commercial or financial relationships that could be construed as a potential conflict of interest.

The reviewer MR declared a past co-authorship with one of the authors CR to the handling Editor, who ensured that the process met the standards of a fair and objective review.

## Footnotes

1 http://microplants.fieldmuseum.org/.
